# Rapid determination of ^237^Np in soil samples by multi-collector inductively-coupled plasma mass spectrometry and gamma spectrometry

**DOI:** 10.1007/s10967-013-2677-6

**Published:** 2013-08-02

**Authors:** Xiaowei Yi, Yanmei Shi, Jiang Xu, Xiaobing He, Haitao Zhang, Jianfeng Lin

**Affiliations:** Northwest Institute of Nuclear Technology, Xi’an, 710024 Shaanxi People’s Republic of China

**Keywords:** ^237^Np, MC-ICP-MS, Gamma-spectrometry, ^239^Np tracer

## Abstract

A radiochemical procedure is developed for the determination of ^237^Np in soil with multi-collector inductively-coupled plasma mass spectrometry (MC-ICP-MS) and gamma-spectrometry. ^239^Np (milked from ^243^Am) was used as an isotopic tracer for chemical yield determination. The neptunium in the soil is separated by thenoyl-trifluoracetone extraction from 1 M HNO_3_ solution after reducing Np to Np(IV) with ferrous sulfamate, and then purified with Dowex 1 × 2 anion exchange resin. ^239^Np in the resulting solution is measured with gamma-spectrometry for chemical yield determination while the ^237^Np is measured with MC-ICP-MS. Measurement results for soil samples are presented together with those for two reference samples. By comparing the determined value with the reference value of the ^237^Np activity concentration, the feasibility of the procedure was validated.

## Introduction

Neptunium-237 (*T*_1/2_ = 2.14 × 10^6^ years) is released to the environment as a result of nuclear weapons tests, reactor accidents and nuclear fuel reprocessing. As a multivalent element, Np may be mobile in certain speciation and migrates into biosphere with underground water. When ingested by human being, Np accumulates in the liver and bones. Therefore, ^237^Np is regarded as a highly radiologically toxic pollutant due to its alpha particle emission and long half-life. In order to assess its environmental risk and determine its origin, the quantification of ^237^Np in soil is necessary. Due to the low concentration of ^237^Np in the environment, preconcentration is usually required before it can be measured with alpha-spectrometry or ICP-MS [1]. ^236^Pu or ^242^Pu is usually used as a yield tracer for ^237^Np because there lack appropriate isotopic tracers for ^237^Np yield determination [2–4]. Isotopes of Pu are not good tracers for Np because the two elements often fractionate during chemical processing. ^235^Np is a potential tracer for ^237^Np [5, 6]. However, ^235^Np free from contamination of ^237^Np is not commercially available. ^236^Np was utilized as the tracer by some researchers to assay ^237^Np in environmental samples where measurement was carried out with mass spectrometry [7–9]. However, ^236^Np is not easy to produce and still not available in pure form to most researchers [4]. ^239^Np has been used as a yield tracer for chemical recovery determination of ^237^Np in the environmental samples with alpha-spectrometry [6, 10–13]. However, the analysis of ^237^Np with alpha-spectrometry usually costs too much time due to its low concentration. Such analytical approaches also require additional chemical operations, such as electrolytic deposition. MC-ICP-MS is particularly effective for measurement of long-lived actinide isotopes with lower specific activity, including ^237^Np. The purpose of this paper is to develop a method for rapid determination of ^237^Np using ^239^Np as a yield tracer, in which MC-ICP-MS and gamma-spectrometer are employed to measure ^237^Np and ^239^Np, respectively.

## Experimental

### Preparation of ^239^Np tracer

**Fig. 1:**
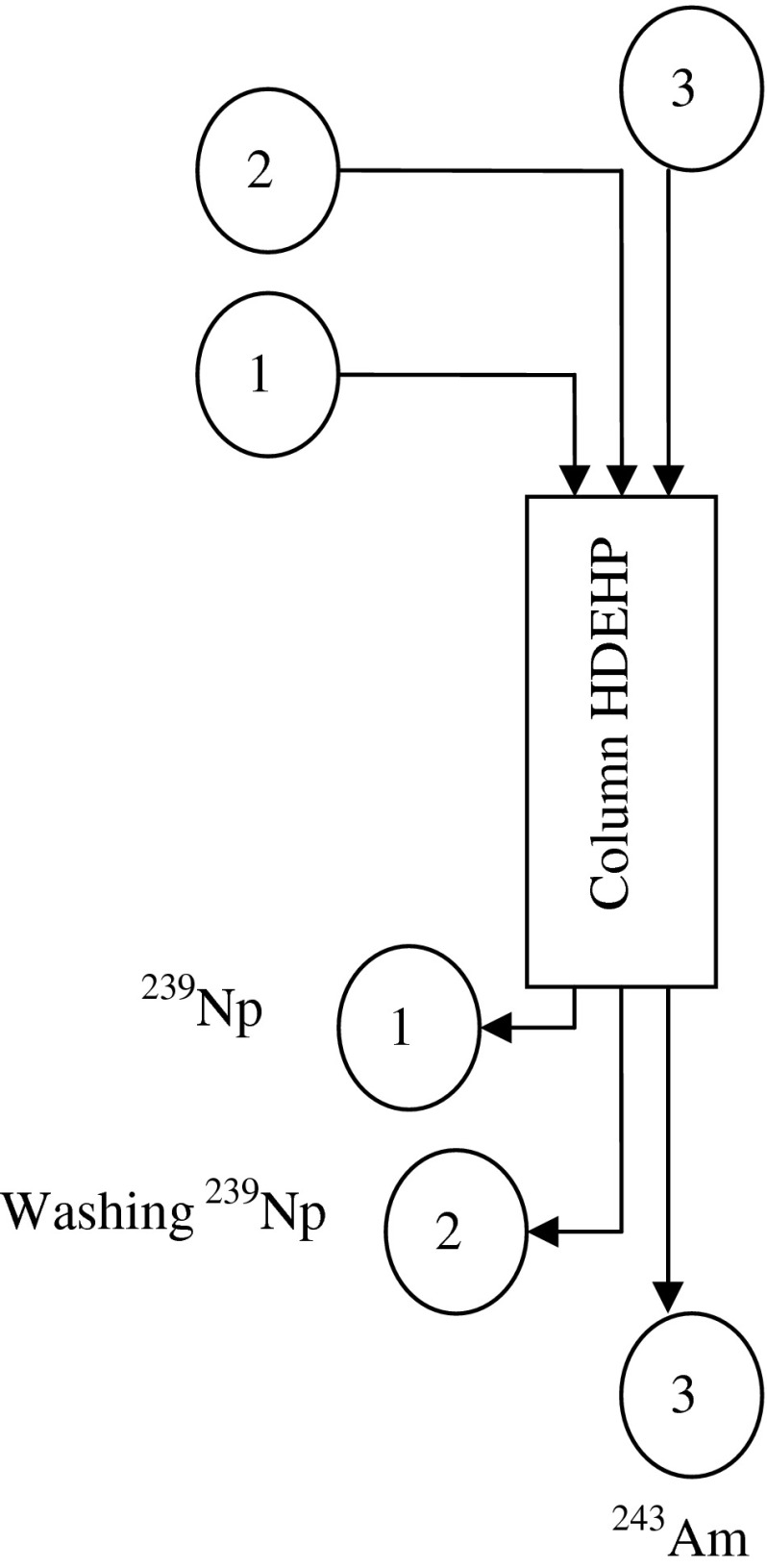
The neptunium-americium separation scheme *1* loading: 0.1 M HNO_3_; *2* rinsing: 0.1 M HNO_3_; *3* elution: 1 M HNO_3_

^239^Np in radioactive equilibrium with its ^243^Am parent was separated with HDEHP extraction chromatography resin (100–200 mesh, Beijing Research Institute of Chemical Engineering and Metallurgy, China) as shown in Fig. 1. Briefly, (1) Add ^243^Am spike solution of ~10^5^ Bq to a beaker and dilute to 0.1 M HNO_3_ with deionized water. (2) The solution is passed through a HDEHP extraction chromatography resin column (0.75 cm i.d. × 10 cm long) which is pre-equilibrated with 15 ml 0.1 M HNO_3_ at a flow rate of 1 ml/min and washed with 10 ml 0.1 M HNO_3_ to improve the recovery of ^239^Np. The eluate is collected into a clean vessel. (3) The eluted ^239^Np in the vessel was measured by gamma-ray spectroscopy to check the yield of this isotope. (4) ^243^Am was later recovered by washing the column with 20 ml of 1 M HNO_3_.

### Procedure for determination of ^237^Np in soil

**Fig. 2:**
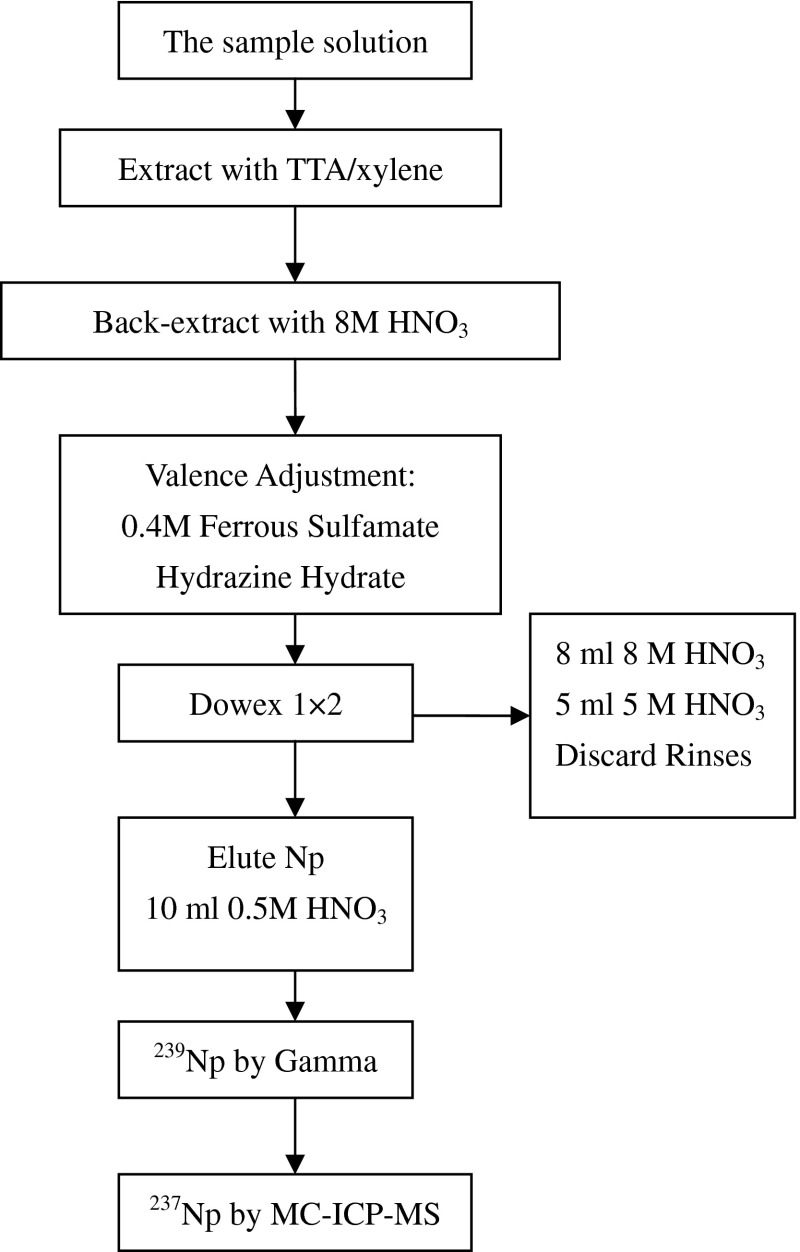
The flowchart of the procedure for determination of ^237^Np

The new procedure for determination of ^237^Np in environmental soil samples is based on thenoyl-trifluoroacetone (TTA) extraction combined with anion exchange chromatography as illustrated in Fig. 2 and described as following.

1. The soil sample was pulverized and dried to constant weight in an oven at 110 °C, and then homogenized with a spatula.
2. Add 1 g of homogenized soil to a clean beaker and ignite at 550 °C in a furnace over night.
3. Transfer the ignited soil sample to a Teflon beaker. Add ^239^Np tracer (about 1000 Bq), 20 ml 15 M HNO_3_, 40 ml 22 M HF and 8 ml 12 M HClO_4_ to the sample and digest the mixture on a hot plate for 2 h until the soil sample is dissolved and the solution is clear. Evaporate the solution to dryness at 150 °C on a hot plate and then transfer the residue to a 100 ml glass beaker. Ignite the residue for 30 min at 550 °C in a furnace.
4. Dissolve the residue with 1 M HNO_3_ solution. Add 1 ml 0.4 M ferrous sulfamate to the solution. Stir it with a glass rod and let it stand for 15 min to reduce Np to IV state completely.
5. The Np(IV) is now extracted from the aqueous HNO_3_ solution by contacting it with 5 ml of a 0.5 M solution of TTA in xylene. Use a mechanical shaker for this operation, with a contact time of 15 min. Repeat this operation once with a fresh TTA-xylene solution and combine the two xylene solutions. Strip the combined xylene solution with 10 ml of 1 M HNO_3_ in the mechanical shaker with a contact time of 10 min. Discard the aqueous phase.
6. Back-extract Np(IV) from the organic phase twice, each with 5 ml 8 M HNO_3_ for 10 min. Combined the aqueous phases and wash with 5 ml xylene for 10 min. Discard the organic phase.
7. Add 1 ml 0.4 M ferrous sulfamate and 0.5 ml hydrazine hydrate to the aqueous solution. Stir it with a glass rod and let it stand for 10 min to reduce Np to IV state completely.
8. The solution is passed through an anion exchange column (0.5 cm i.d. × 10 cm long) containing Dowex 1 × 2 resin (100–200 mesh, Sigma-aldrich, USA). The resin in the column must be pretreated by passing 10 ml 8 M HNO_3_ through it with a flow rate of 0.2 ml/min. Wash the column with 8 ml 8 M HNO_3_ and 5 ml 5 M HNO_3_ successively to further remove U and other elements in the soil matrix.
9. Strip Np with 10 ml 0.5 M HNO_3_ and evaporate the eluate solution to dryness at 150 °C on a hot plate.
10. The sample is dissolved with 3 ml 0.1 M HNO_3_ and transferred into a vial for gamma-spectrometric measurement of chemical yield.
11. The quantity of ^237^Np was measured with MC-ICP-MS using the standard addition method. After the 3-ml solution of 0.1 M HNO_3_ is nondestructively analyzed for ^239^Np by gamma-ray spectrometry, it is divided into six equal aliquots. Five of these aliquots are individually added to five standard solutions of ^237^Np. The sixth aliquot is added to an equal volume of a solution containing no ^237^Np. All six solutions are immediately analyzed for ^237^Np by MC-ICP-MS.

## Results and discussion

### Purity check of ^239^Np tracer

**Fig. 3:**
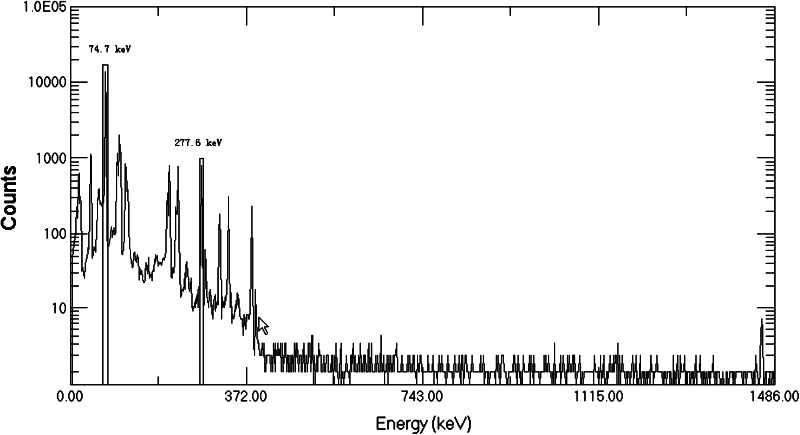
Gamma-spectrum of ^243^Am solution in equilibrium with ^239^Np

**Fig. 4:**
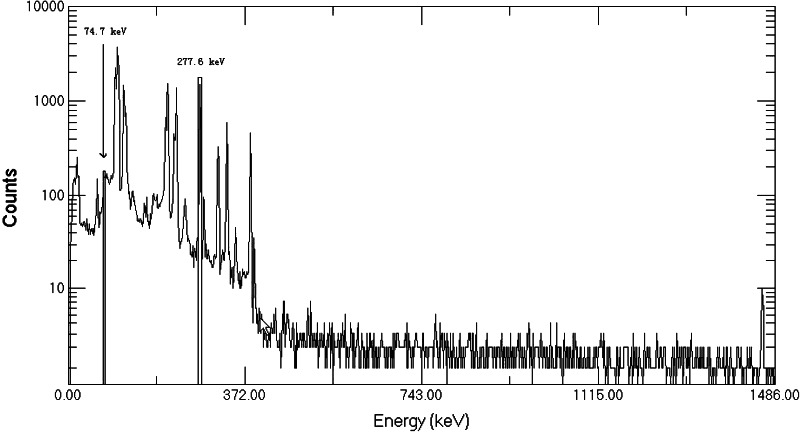
Gamma-spectrum of purified ^239^Np

The ^239^Np intended as a tracer for ^237^Np was obtained from ^243^Am as described above. In order to assure the suitability of the ^239^Np as a tracer, its purity was checked with a HPGe gamma-spectrometer. Figure 3 shows the gamma-ray spectrum of ^243^Am in equilibrium with ^239^Np before separation. The peaks of both ^239^Np (277.6 keV) and ^243^Am (74.7 keV) appear clearly in the spectrum. However, when ^239^Np was separated from ^243^Am parent and checked with a HPGe gamma-spectrometer, only the peak of ^239^Np can be seen in the spectrum and no lines for ^243^Am are distinguishable in the spectrum (see Fig. 4).

^239^Np is a short-lived β-emitter (*T*_1/2_ = 2.355 days) while ^243^Am is a long-lived α-emitter (*T*_1/2_ = 7370 years). The activity equilibrium of this mother/daughter pair is reached after 23.6 days, about 10 half lives of ^239^Np [14, 15]. Therefore, ^239^Np can be prepared from the same ^243^Am solution again and again after the activity equilibrium of this mother/daughter pair is reached.

### Validation of the analytical procedure

In order to validate the applicability of the analytical procedure to soil samples with complicated matrix, two reference soil samples R1 (1 g) and R2 (1 g), with known ^237^Np activity concentration were analyzed for ^237^Np concentration according to the procedure described above and the results are compared with the reference values in Table 1. The ^237^Np activity concentration of R1 was 0.040 Bq/g, with a difference of −3.6 % compared to the reference value. The ^237^Np activity concentration of R2 was 0.050 Bq/g, with a difference of 2.0 % compared to the reference value. It can be seen that the determined results are in good agreement with the reference data for both reference soils, suggesting that the new analytical method applies to soil sample very well.

**Table 1:** Results of ^237^Np concentration determination of reference soils

Sample no.	Determined value (Bq/g)	Reference value (Bq/g)	Difference (%)
R1	0.040 ± 0.002	0.041	−3.6
R2	0.050 ± 0.003	0.049	2.0

### Application of the proposed procedure in practical environment samples assay

To verify the feasibility of the proposed analytical procedure for practical environment samples, the ^237^Np activity concentrations of the two real soil samples (namely, S1 and S2) were determined following the proposed procedure. The S1 and S2 surface soil samples (sandy soil) were collected from the northwest of China, near a nuclear facility site. The soils were ground and sieved to get a powder with particle diameters ranging from 74 to 149 μm. The uniformity was checked with gamma spectrometric measurement.

After the separation procedure, the resulting solutions containing purified ^237^Np and spiked ^239^Np were transfered to clean tubes and measured with the gamma-spectrometer in the same geometry as the ^239^Np tracer solution had been measured. By this way, the detection efficiency of the detector is not required to be calibrated because only relative counts in the same equipment are used in calculations. The 277.6 keV photopeak of ^239^Np was chosen because of its relatively high intensity and its location in a comparatively flat baseline region of the spectrum. The yield of ^239^Np (denoted *Y*) is calculated by means of Eq. (1).

1 $$ Y = C_{\text{s}} /(C_{0} e^{ - \lambda t} ) $$

where *C*_s_ is the counts of ^239^Np in the resulting solution, cps; *C*_0_ is the counts of ^239^Np in added tracer spike, cps; *λ* is the the decay constant of ^239^Np, s^−1^; *t* the time gap between the two measurements of ^239^Np, s.

The yields of ^239^Np are listed in Table 2 together with the determined ^237^Np concentration. It can be seen that although the ^237^Np activity concentration of the two soil samples is very low, the reproducibility of the results is <6 %, which is rather good for environmental analysis. However, the chemical yield is a bit lower due to the complicated matrix of the soil.

**Table 2:** Results of ^237^Np determinations of soils using ^239^Np tracer

Sample ID.	Soil mass (g)	^239^Np yield (%)	^237^Np activity concentration (Bq/g)	Difference between duplicates (%)	Average^237^Np activity concentration (Bq/g)
S1-1	1.0512	28	0.055	2.3	0.056
S1-2	1.1134	30	0.057		
S2-1	1.0234	30	0.028	5.5	0.029
S2-2	1.1206	22	0.030		

Due to the low concentration of ^237^Np in the soil samples, the measurement with alpha-spectrometry [13] may cost too much time (about 3–4 days) in order to have precise results. Besides, the matrix components, including interfering elements and radionuclides, have to be removed effectively with many difficulties for α measurement. With this new analytical method, it takes 2 days less for measurement with MC-ICP-MS [13]. Methods that use ^242^Pu [3, 4] as a tracer for ^237^Np presume that the chemistries of Pu and Np are so similar that Pu and Np follow one another in soil and in the chemical treatment of soil samples. No such assumption is made in the new method because ^239^Np and ^237^Np have identical chemistries.

## Conclusion

A new rapid separation method was developed for the determination of ^237^Np in soil samples with MC-ICP-MS and gamma-spectrometry. There are two advantages of the present procedure. One is that it adopts gamma-emitting ^239^Np instead of ^236^Pu or ^242^Pu as the tracer, and the other is that the yield of Np can be determined without relative efficiency calibration of gamma-spectrometer for ^239^Np sources. The feasibility of the procedure was validated by analyzing the two reference soil samples with known ^237^Np activity concentration.
